# Utility of Whole-Genome Sequencing to Ascertain Locally Acquired Cases of Coccidioidomycosis, Washington, USA

**DOI:** 10.3201/eid2503.181155

**Published:** 2019-03

**Authors:** Hanna N. Oltean, Kizee A. Etienne, Chandler C. Roe, Lalitha Gade, Orion Z. McCotter, David M. Engelthaler, Anastasia P. Litvintseva

**Affiliations:** Washington State Department of Health, Shoreline, Washington, USA (H.N. Oltean);; Centers for Disease Control and Prevention, Atlanta, Georgia, USA (K.A. Etienne, L. Gade, O.Z. McCotter, A.P. Litvintseva);; Translational Genomics Research Institute, Flagstaff, Arizona, USA (C.C. Roe, D.M. Engelthaler)

**Keywords:** Coccidioides, coccidioidomycosis, valley fever, fungi, fungal infections, whole-genome sequencing, surveillance, public health, Washington, United States

## Abstract

Coccidioidomycosis is an emerging fungal infection in Washington, USA, and the epidemiology of the disease in this state is poorly understood. We used whole-genome sequencing to differentiate locally acquired cases in Washington on the basis of the previously identified phylogeographic population structure of *Coccidioides* spp. Clinical isolates from coccidioidomycosis cases involving possible Washington soil exposure were included. Of 17 human infections with epidemiologic evidence of possible local acquisition, 4 were likely locally acquired infections and 13 were likely acquired outside Washington. Isolates from locally acquired cases clustered within the previously established Washington clade of *C. immitis*. Genetic differences among these strains suggest multiple environmental reservoirs of *C. immitis* in the state.

Coccidioidomycosis, also known as Valley fever, is a disease of growing public health concern and is caused by 2 closely related fungal species, *Coccidioides immitis* and *C. posadasii*. *Coccidioides* spp. are dimorphic, forming mycelia in soil and arthroconidia capable of infecting humans and certain other mammals, and spherules in mammalian tissue (*1*). This mycosis causes a wide spectrum of conditions, ranging from asymptomatic infection and mild pulmonary disease to severe pulmonary and disseminated disease, which can be life-threatening (*1**,**2*). Infection is generally caused by inhalation of pathogenic arthroconidia from disturbed soil or dust, such as through occupational or recreational activities or weather events that raise dust (*2**–**7*).

*Coccidioides* spp. are endemic to warm, arid regions of the Western Hemisphere that have low rainfall (*1**,**8*). The 2 species have colonized different geographic locations: *C. posadasii* is largely found in Arizona, Texas, Mexico, and Central and South America; and *C. immitis* is primarily found in central and southern California. Coccidioidomycosis causes a major burden of disease in many of these areas; the annual incidence in Arizona is >75 cases/100,000 population (*9*). Case counts have recently increased in California; >5,300 cases were reported in 2016 (13.7 cases/100,000 population (*10*). Climate suitability projections predict expansion of the suitable environment for coccidioidomycosis and increasing incidence in areas that already sustain *Coccidioides* growth (*11*).

During 2010–2011, three cases of coccidioidomycosis were identified and reported in southeastern Washington (*7*). Subsequently, *Coccidioides* DNA and viable propagules were isolated from soil at a location of suspected exposure for 1 of these cases (*12**,**13*). Whole-genome sequencing (WGS) analysis of the recovered isolates demonstrated near identical genetic identity between soil and clinical isolates, further confirming endemic presence of infectious *C. immitis* in Washington (*13*).

Public health surveillance for coccidioidomycosis was implemented statewide in Washington in April 2014. Before 2014, cases of coccidioidomycosis were reported sporadically, but no standard reporting procedure existed. Since 2014, the number of reported cases of coccidioidomycosis has increased each year; reported cases in Washington suspected to be locally acquired have higher rates of hospitalization and death compared with cases from other disease-endemic regions (*14*).

Because most persons infected with *Coccidioides* spp. are asymptomatic or have only mild illness, and most illness self-resolves, we believe that only the most severe cases of coccidioidomycosis with exposure in Washington are being identified and reported to public health authorities. In addition, *Coccidioides* spp. infections might be asymptomatic or only manifest as subclinical disease until a reactivation or complication develops later. Also, most residents of Washington have some travel history to historically disease-endemic areas, such as Arizona, California, or Mexico. These findings create a challenge in differentiating between autochthonous and imported cases of coccidioidomycosis, which is a useful distinction for public health surveillance in Washington.

To better determine the nature of exposure for reported cases of coccidioidomycosis in Washington, the Washington Department of Health, in collaboration with the Centers for Disease Control and Prevention (CDC; Atlanta, GA, USA) and the Translational Genomics Research Institute (Flagstaff, AZ, USA), initiated enhanced surveillance by incorporating WGS of clinical isolates aimed at determining the geographic origins of *Coccidioides* spp. strains. This approach is based on previous population structure studies providing evidence that *C. immitis* and *C. posadasii* have well-defined geographic structures (*15**–**19*). Subsequently, Engelthaler et al. demonstrated that isolates can generally be assigned to the specific geographic populations on the basis of WGS analysis (*20*). This approach is consistent with a general trend in molecular epidemiology that uses WGS and a One Health approach to ascertain clusters or outbreaks of bacterial, fungal, viral, and parasitic diseases (*21**–**23*).

We report WGS analyses of isolates from 17 recent coccidioidomycosis cases in Washington, and demonstrate that 13 (76%) cases involved coccidioidomycosis most likely acquired outside Washington; 4 cases (24%) likely involved local acquisition. We discuss the utility of the WGS method to enhance epidemiologic surveillance.

## Materials and Methods

### Cases and Isolates

We identified coccidioidomycosis cases through passive reporting to local health jurisdictions from healthcare providers in Washington and laboratories performing testing for residents of Washington. Confirmed cases, as classified according to the Council of State and Territorial Epidemiologists case definition (*24*), reported during 2014–2017 were included in the analysis. Reported case-patients were interviewed by local health jurisdictions to determine clinical course, travel history, and any potential soil or dust exposures. Medical records were requested when possible for complete data abstraction to case reporting forms. When the exposure was suspected to have occurred in the disease-endemic area of Washington or exposure history was unknown, available clinical isolates were sent to CDC for confirmation.

At CDC, all isolates were grown on brain heart infusion (BHI) agar at 25°C for 10 days. Genomic DNA was extracted by using the DNeasy Blood and Tissue kit (QIAGEN, https://www.qiagen.com/us) according to the manufacturer’s recommendations. Genomic DNA was stored at −20°C until further use. The Washington State and CDC Institutional Review Boards determined this project to be enhancing surveillance for a notifiable condition and did not require human subjects review.

### Genome Sequencing, Assembly, and Analyses

We sequenced genomes of 18 isolates from 17 patients using Illumina (https://www.illumina.com) HiSeq and MiSeq sequencing platforms, as described (*20**,**25*). We prepared DNA samples for paired-end sequencing by using the Kapa Biosystems (https://www.kapabiosystems.com) Library Preparation Kit protocol with an 8-bp index modification and sequenced to a read length of 250 bp on the Illumina HiSeq or 250 bp on the Illumina MiSeq. All WGS data files have been deposited in the National Center for Biotechnology Information Sequence Read Archive under BioProject PRJNA472461.

We performed genome assembly and analyses as described (*20*). In brief, the Washington-1(B10637) strain was used as the reference for *C. immitis* single-nucleotide polymorphism (SNP) matrices, and the B10813_Tx strain was used as a reference in the *C. posadasii* SNP matrix. We aligned Illumina read data against the respective reference assemblies by using Burrow-Wheeler Aligner version 0.7.7 (http://bio-bwa.sourceforge.net) and identified SNP variants by using SamTools version 0.1.19 (http://samtools.sourceforge.net). We filtered SNP calls by using a publicly available SNP analysis pipeline (*26*) (http://tgennorth.github.io/NASP), as described, to remove positions with <10 times coverage with <90% variant allele calls, or identified by using Nucmer (http://nebc.nox.ac.uk/bioinformatics/docs/nucmer.html) as being within duplicated regions in the reference (*20**,**25*). We constructed a maximum-parsimony phylogeny on the basis of SNP matrices by using MEGA7 (http://www.megasoftware.net), including 17 described strains from various geographic areas for comparison; we determined bootstrap support for the tree by using 1,000 reiterations (*20*).

We extracted candidate SNPs that distinguish Washington strains from non-Washington strains (Appendix Table) from whole-genome assemblies by using Samtools version 1.8, EMBOSS version 6.5.7, and NCBI-toolkit version 7.0.0 (*27**–**29*). We chose SNPs if their positions were consecutive or no more than 1 nt apart and had >10 times coverage. In brief, we extracted an ≈1-kb region surrounding candidate SNP positions from all draft assemblies, aligned it to Washington-1(B10637) reference, and designed degenerate primers by using Primer3 version 3.0 (*30*). We compared regions with those of *C. immitis* strain H538–4 to determine genomic loci annotation.

## Results

During 2014–2017, a total of 167 confirmed cases of coccidioidomycosis were reported in Washington. For these cases, 18 isolates (Table) from 17 cases were available. Travel and exposure histories suggested local acquisition in Washington (9 case-patients) or were unknown at the time of specimen collection (8 case-patients).

**Table:** Characteristics of 18 clinical isolates subjected to whole-genome sequencing analysis from patients with coccidioidomycosis, Washington, USA, 2014–2017*

CDC-ID	Travel history of patient	*Coccidioides *species	Collection date	Specimen source site	Phylogenetic population
B10917	Unknown	*C. posadasii*	2014	Sputum	Non-Washington
B10918	AZ, MT, SD, OR	*C. posadasii*	2014	Sputum	Non-Washington
B11036	AZ	*C. posadasii*	2015	Lung	Non-Washington
B11034	AZ	*C. immitis*	2014	Lung	Washington
B11019	None	*C. immitis*	2014	BAL	Washington†
B11035	CA	*C. immitis*	2015	Cerebrospinal fluid	Non-Washington
B11080	Mexico, CA	*C. immitis*	2015	Neck	Non-Washington
B11198	Mexico, CA	*C. immitis*	2015	Tissue	Non-Washington
B11299	AZ	*C. posadasii*	2015	Bronchial washings	Non-Washington
B11517	CA	*C. immitis*	2016	Throat	Non-Washington
B11518	None	*C. immitis*	2016	Testicle	Washington†
B11587	CA	*C. immitis*	2016	Tissue	Non-Washington
B11863	HI, Costa Rica	*C. immitis*	2016	Sputum	Non-Washington
B11873	CA	*C. immitis*	2016	Bronchial washings	Non-Washington
B12398	None	*C. immitis*	2016	Blood	Washington
B12495	CA	*C. immitis*	2016	Cerebrospinal fluid	Non-Washington
B12496	Mexico, CA	*C. immitis*	2016	Knee wound	Non-Washington
B13956	None	*C. immitis*	2017	BAL	Washington

*BAL, bronchoalveolar lavage; CDC, Centers for Disease Control and Prevention; ID, identification. †These isolates were from the same patient.

We speciated 14 isolates as *C. immitis* and 4 as *C. posadasii* and subjected these isolates to WGS with 25–160 times sequencing coverage. A total of 68,717 SNPs were identified by comparison with the reference isolate. Phylogenetic analysis and comparison with sequenced isolates of *C. immitis* demonstrated that 5 isolates from 4 patients in Washington were highly genetically related to each other and to isolates from the identified Washington clade that contained human and soil isolates (*13*). No more than 354 SNPs differentiated any 2 isolates within the Washington cluster, which was also supported by 100% bootstrap values (Figure). In contrast, >10,000 SNPs differentiated the Washington cluster from its nearest neighbor, an isolate from another case-patient in Washington. This case-patient (B12496) reported travel history to disease-endemic regions in California and Mexico. Tens of thousands of SNPs differentiated Washington cluster isolates from those from other states. No more than 3 SNPs were identified among multiple soil isolates collected 4 years apart from a single sampling site, and 4 SNPs were identified between isolates collected 2 years apart from 1 patient in Washington who had a chronic infection. However, 234–324 SNPs were identified in pairwise comparisons between isolates from different patients in the Washington clade (Figure, panel B).

**Figure:**
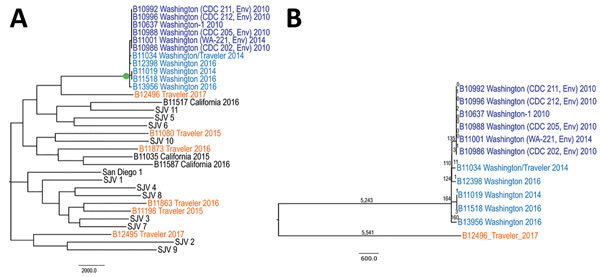
Genetic relationships among *Coccidioides immitis* isolates. A) Isolates from patients in Washington, USA, compared with isolates from other locations. B) Genetic relationships among *C. immitis* isolates from the Washington clade; single-nucleotide polymorphism numbers are shown above the branches. Dark blue indicates previously described environmental and human isolates (*13*) from Washington, light blue indicates isolates from new cases that were likely acquired in Washington, and orange indicates isolates from cases that were likely acquired outside Washington. Isolates from patients with the documented travel histories are indicated as Traveler. Travel history is detailed in the Table. Isolates with SJV names are from the San Joaquin Valley, California, as described (*20*). Bootstrap values shown in green circle indicate 100% support. Scale bars indicate nucleotide substitutions per site. CDC, Centers for Disease Control and Prevention; Env, environment.

Three of the 4 case-patients infected with Washington clade strains reported no travel history outside Washington. Conversely, 8 of 9 *C. immitis* isolates that did not cluster with the Washington clade were isolated from patients who reported travel to California.

Washington strains can be distinguished from non-Washington strains in 3 genomic regions on the basis of annotations from *C. immitis* strain H538-4; locus DS016982: adipocyte-derived leucine aminopeptidase; locus DS01702: hypothetical protein; and locus DS016985: glyoxal oxidase (Appendix). The DNA fragment from locus DS016982 contains SNP GG at positions 94460–94461 for Washington strains and SNP CA for non-Washington strains and can be amplified by using forward primer 5′-GGTACGTCACAAGTCCCCAG-3′ and reverse primer 3′-AAGAGTACTCGCGAAGGAAGC-5′. For locus DS017021, the DNA fragment amplified with forward primer 5′-CTTGACTGTGCAGGGCCTTA-3′ and reverse primer 3′-ACCGGCCTAACTCCATGGTA-5′ encompasses SNPs GGT for Washington strains and TGC for non-Washington strains at positions 105566–105568) The fragment of locus DS016985 can be amplified by using primer pairs 5′-TTCCGCTTGATGGCTGAAGT-3′/3′-TGTGGCCCTCCTATTGCTTG-5′ and contains consecutive SNP CC for Washington strains and SNP GA for non-Washington strains (positions 232442–232443).

We identified 4 case-patients who had *C. posadasii* infections by using internal transcribed spacer sequencing and confirmed by WGS. Isolates from the 4 case-patients clustered with described *C. posadasii* populations from Arizona (data not shown). Three of those case-patients had traveled to Arizona, and 1 case-patient had an unknown travel history.

## Discussion

Determining the potential sources of exposure and geographic distribution of *Coccidioides* in areas where coccidioidomycosis is uncommon is a surveillance challenge because patients often report travel histories to other disease-endemic areas. To address this problem, we implemented WGS to better determine the likely geographic origin of isolates obtained from patients in Washington who had suspected local acquisition or unknown exposure histories. This method is based on the observation that *Coccidioides* populations have well defined geographic structure, and most isolates can be assigned to specific geographic areas on the basis of their WGS genotypes, such as Arizona, Texas, Mexico, central California, or Washington (*20*). We demonstrated the utility of this tool for investigating the epidemiology of coccidioidomycosis in Washington.

Of 18 isolates from 17 patients included in this study, 5 (28%) clustered with strains isolated from soil in Washington, which was consistent with local exposure. The remaining 13 (72%) isolates clustered with populations from other areas, such as Arizona, California, or Mexico, which was generally consistent with reported travel histories of patients. The only discordance between WGS and epidemiologic data was observed for isolate B11863, which clustered with *C. immitis* isolates from California. The patient from whom this isolate originated reported travel out of state but also reported extensive soil exposure in a county in Washington that had previously reported autochthonous cases. Specifically, the only reported travel was to Hawaii 2 months before disease onset and to Costa Rica 1 year before disease onset, with a layover in California during which the patient did not leave the airport. In addition, the patient reported 1 trip to southern California 6 years before disease onset. This discordance was an unexpected finding and might represent more diversity among *Coccidioides* spp. in Washington than previously understood, reactivated infection because of previous travel, or infection from a fomite or other carrier source of *C. immitis*. Further investigation and surveillance data are needed to determine which hypothesis is most plausible.

Of the 4 patients infected with the local strains from Washington, 3 patients reported no travel history outside Washington. The fourth patient reported travel to Arizona 1 year before the onset of coccidioidomycosis but also reported extensive exposure to local soils in Washington and dust during hiking and gardening. This result indicates that compatible travel history alone does not exclude the possibility of local exposure and demonstrates the added value of genomic approaches to determining exposure location. Although isolates in the Washington clade were genetically related (Figure, panel A), considerably higher genetic diversity was observed for new strains compared with that detected for the original isolates from Washington identified in 2013 (*13*). Specifically, 245–299 pairwise SNPs were identified among the newly identified strains from Washington, compared with <5 SNPs detected among previously described isolates from Washington from the same exposure site (Figure, panel B). This small but apparent genetic divergence of the new isolates indicates that new infections were acquired from different local *C. immitis* populations, likely at different sites, and suggests the presence of multiple environmental loci for *Coccidioides* spp. in Washington. The divergent isolates were collected from soil in a location ≈70 miles from the suspected exposure location of 1 of the patients. However, in sharp contrast to the populations of *Coccidioide*s spp. in Arizona or California, where isolates are differentiated by thousands of SNPs, the overall low genetic variability in the Washington population is consistent with a relatively small population size, strongly suggesting a recent common ancestry of Washington strains and a relatively recent expansion of *C. immitis* to Washington, (Figure).

Our study has 2 main limitations. First, there might be additional genetically diverse populations of *Coccidioides* in Washington that have not yet been discovered or proven; therefore, some genotypes that have been deemed as acquired outside Washington might have been acquired locally. For example, 1 isolate from a patient who was highly suspected of having local acquisition clustered with isolates from California. Likewise, there might be genetically similar populations of *Coccidioides* spp. in Oregon, California, or other nearby states that have not yet been documented. These genetically similar populations might add complexity to determination of location of exposure. However, to date, no clinical or environmental isolates with strong epidemiologic links to locations outside Washington that cluster with isolates from Washington have been identified. This finding is consistent with those of previous studies that demonstrated that *C. immitis* and *C. posadasii* have well-defined geographic structures (*15**–**19*).

Second, all patients in our study were given diagnoses of rather atypical cases of coccidioidomycosis that were either asymptomatic (diagnosed during biopsy), cutaneous, chronic, or unusually severe. In general, the acute primary pulmonary form of coccidioidomycosis constitutes the most common manifestation in highly endemic areas and often represents the earliest manifestation of the disease. Conversely, reactivation of latent infections can occur months to years after initial exposure, typically in immunosuppressed persons. Many complicated infections might follow a subacute or chronic disease progression. This lack of acute respiratory cases and preponderance of atypical disease in Washington indicates the likely gap between early identification and delayed diagnosis because of lack of awareness and might also explain the high prevalence of the out-of-state strains of *C. immitis* in our study. However, even in highly endemic regions, acute pulmonary disease is often missed or delayed in diagnosis for months (*31**,**32*), supporting the possibility that an underlying background of typical acute disease might remain unidentified in Washington (*33*).

Our results indicate that WGS is a useful tool to assist in determining exposure location in surveillance situations in which exposure histories are unclear or unknown. For patients in this study, coccidioidomycosis was more commonly associated with travel to other disease-endemic areas compared with local exposure; however, travel to endemic regions does not preclude local acquisition of the disease. Our results also indicate that coccidioidomycosis is likely to be underdiagnosed and underreported in Washington on the basis of atypical disease manifestations for locally acquired cases. More research is needed to determine the true prevalence of locally acquired coccidioidomycosis in Washington.

Appendix Additional information on utility of whole-genome sequencing to ascertain locally acquired cases of coccidioidomycosis, Washington, USA.
