# Supplementary material for: Utility of Whole-Genome Sequencing to Ascertain Locally Acquired Cases of Coccidioidomycosis, Washington, USA
Source: Emerg Infect Dis. 2019 Mar;25(3):501–6. doi: 10.3201/eid2503.181155 (PMC6390764; doi:10.3201/eid2503.181155)
Supplement: Appendix — Additional information on utility of whole-genome sequencing to ascertain locally acquired cases of coccidioidomycosis, Washington, USA. [file 18-1155-Techapp-s1.pdf]

Article DOI: https://doi.org/eid2503.181155

Utility of Whole-Genome Sequencing to Ascertain Locally Acquired Cases of Coccidioidomycosis, Washington, USA

Appendix

Appendix Table. Genomic SNPs that distinguish Washington of strains of *Coccidioides immitis* from non-Washington strains*

Locus ID	Annotation	Position	Forward primer, 5′→3′	Reverse primer, 3′→5′	Washington strain SNPs	Non-Washington strain SNPs
DS016982	Adipocyte-derived leucine aminopeptidase	94460–94461	GGTACGTCACAAGTCCCCAG′	AAGAGTACTCGCGAAGGAAGC	GG	CA
DS017021	Hypothetical protein	105566–105568	CTTGACTGTGCAGGGCCTTA	ACCGGCCTAACTCCATGGTA	GGT	TGC
DS016985	Glyoxal oxidase	232442–232443	TTCCGCTTGATGGCTGAAGT	TGTGGCCCTCCTATTGCTTG	CC	GA

*Genomic annotations are based on *C. immitis* H538–4. ID, identification; SNP, single-nucleotide polymorphism.
